# Long Non-coding RNA AK025387 Promotes Cell Migration and Invasion of Gastric Cancer

**DOI:** 10.3389/fonc.2020.00633

**Published:** 2020-05-20

**Authors:** Yi-Yuan Sun, Hui Zhang, Ran-Ran Ma, Guo-Hao Zhang, Ya-Ru Tian, Lei Liu, Lin Liu, Peng Gao

**Affiliations:** ^1^Department of Pathology, Qilu Hospital, Shandong University, Jinan, China; ^2^Key Laboratory for Experimental Teratology of the Ministry of Education, Department of Pathology, School of Basic Medical Sciences, Cheeloo College of Medicine, Shandong University, Jinan, China

**Keywords:** long non-coding RNA, gastric cancer, AK025387, migration and invasion, MAPK signaling pathway

## Abstract

Gastric cancer is one of the most common cancers in the world, and long non-coding RNAs (lncRNAs) play a crucial role in proliferation, metastasis, and invasion of gastric cancer. However, there are very few researches focusing on the effects of lncRNAs on metastatic gastric cancer. In this research, we identify one kind of lncRNA, called AK025387, which is highly expressed in metastatic gastric cancer samples compared with non-metastatic gastric cancer samples. The expression of AK025387 is significantly positively correlated with lymph node metastasis. The *in situ* hybridization demonstrates that AK025387 is located in both nucleus and cytoplasm, but mostly in cytoplasm. AK025387 promotes gastric cancer cells migratory and invasive ability, but it inhibits apoptosis *in vitro*. Furthermore, AK025387 regulates Raf-1, mitogen-activated protein kinase/extracellular signal-regulated kinase (MEK), and extracellular signal-regulated kinase (ERK) and is involved in mitogen-activated protein kinase (MAPK) signaling pathway to perform its biological functions. We conclude that AK025387 is highly expressed in metastatic gastric cancer, and its biological functions suggest the potential of AK025387 to be a biomarker of metastatic gastric cancer.

## Introduction

Gastric cancer is the fifth most commonly diagnosed cancers and the third most common cause of cancer death worldwide (1). Despite the global downward trends in gastric cancer mortality rates, further efforts are still needed (2). The two main features of gastric cancer, tumor invasion and metastasis, cause poor prognosis (3). Therefore, proposition of new treatments via the exploration of the molecular mechanisms of gastric cancer tumorigenesis and development, especially invasion and metastasis, are stringent.

Long non-coding RNAs (lncRNAs) are defined as transcriptional products that are composed of more than 200 nucleotides in length with little or no protein-coding potential (4). LncRNAs were initially thought to be spurious transcriptional noise with little biological function. However, widespread functionality of lncRNAs has been discovered and investigated in recent studies, and some new methods to research lncRNAs were used (5, 6). LncRNAs regulate gene expression at various levels, such as transcriptional regulation, post-transcriptional, protein localization, and RNA interference (7–10). Aberrant lncRNA expression is also involved in tumor proliferation, invasion, and metastasis process (11). For example, the lncRNA Hox transcript antisense intergenic RNA (HOTAIR) promotes migration and invasion of gastric cancer cells by performing as a competing endogenous RNA to regulate HER2 expression (12). In this research, we first identify an lncRNA called AK025387, which is overexpressed in metastatic gastric cancer sample. Then, we investigate migration, invasion, proliferation, and apoptosis ability of gastric cancer cells by downregulating or upregulating AK025387 level. We further explore underlying signaling pathways of AK025387 in gastric cancer cells.

## Materials and Methods

### Human Gastric Cancer Samples

Fresh-frozen tissues of 37 metastatic and 33 non-metastatic gastric cancers between 2013 and 2014 were obtained from the Qilu Hospital of Shandong University. All samples were collected for RNA expression analysis. Methods were performed according to the approved guidelines. All the participants have provided informed consents. The research was approved by the Ethical Committee of Shandong University, China.

### Real-Time Quantitative PCR

Total RNA was extracted using TRIzol (Invitrogen, Carlsbad, CA, USA), according to the manufacturer's recommended protocol, and was reverse transcribed into complementary DNA (cDNA) using a Rever Tra Ace qPCR RT Kit (Toyobo, Osaka, Japan). SYBR Green Real-Time PCR Master Mix (Roche Diagnostic GmbH, Mannheim, Germany) and Applied Biosystems 7900HT were used to perform the real-time quantitative PCR. The relative expression of RNAs was standardized by glyceraldehyde 3-phosphate dehydrogenase (GAPDH).

### Cell Culture and Transfection

The human gastric cancer cell lines MKN45 and SGC7901 were obtained from the Shanghai Cancer Institute. Gastric cancer cells were grown in Roswell Park Memorial Institute (RPMI) 1640 supplemented with 10% fetal bovine serum (FBS, Gibco BRL, Grand Island, NY, USA). X-tremeGENE transfection reagent (Roche Applied Science, Indianapolis, IN, USA) was used to transfect small interfering RNA (siRNA) (siRNA target sequence: GCTATCATTTCCCAGGTTT) or the negative control (RiboBio, Guangzhou, China) into gastric cancer cells according to the manufacturer's instructions. The pcDNA3.1 and pcDNA3.1-AK025387 plasmids (BioSune, Shanghai, China) transfected gastric cancer cells using TurboFect transfection reagent (Thermo Scientific, Shanghai, China) according to the manufacturer's instructions.

### RNA *in situ* Hybridization

The digoxin-labeled *in situ* hybridization (ISH) probe used for detecting AK025387 was designed and synthesized by BioSune Co., Ltd. (Shanghai, China). Cells were seeded on polylysine-processed coverslips. The fresh-frozen tissue was cut at a thickness of 4 μm per section in a cryostat microtome and then was fixed on coverslips with formaldehyde. Coverslips were processed using the Enhanced Sensitive ISH Detection Kit I (POD) (cat: MK1030; Boster, Wuhan, China) according to the manufacturer's protocol. The Biotin-Mouse Anti-Digoxin was used after hybridization. The cell coverslips were visualized with fluorescein isothiocyanate (FITC)-streptavidin–biotin complex (SABC) (Boster, Wuhan, China) for probe and 4′,6-diamidino-2-phenylindole (DAPI) for nucleus. The positive results were stained in green and visualized under a fluorescent microscope (Olympus, Japan). The tissue-frozen sections were processed with SABC and biotin peroxidase and then visualized with 3,3′-diaminobenzidine (DAB) stain for probe and hematoxylin for nucleus. The positive results were stained in yellow.

### RNA Stability Assay

The cells were grown in 12-well plates (Corning, NY, USA) until adherence. All cells were treated with Actinomycin D (Solarbio, Beijing, China) for 0 h, 30 min, 1 h, and 4 h and collected for real-time quantitative PCR (RT-qPCR). C-myc level was used as control.

### Cell Migration and Invasion Assays *in vitro*

Migration assays were performed in Transwell chambers inserts with 8.0-μm pore size membrane (24-well plate, Coster, Corning Inc., Corning, NY, USA), and in invasion assays, membranes of Transwell chambers were coated with the Matrigel matrix (BD Science, Sparks, MD, USA). Gastric cancer cells (loss-of-function experiment, 8 × 10^4^ in migration assays and 1.2 × 10^5^ in invasion assays; gain-of-function experiment, 6 × 10^4^ in migration assays and 1 × 10^5^ in invasion assays) in 200 μl RPMI 1640 media were transferred into the upper chamber 24 h after transfection, and 600 μl RPMI 1640 media with 10% FBS was transferred into the lower chamber. Then, the 24-well plate was transferred into a CO_2_ incubator at 37°C for 24 h. The non-migrating or non-invading cells stayed on the upper surface of the membrane, while the migrated or invaded cells reached the lower surface. The cotton swabs were used to remove non-migrating or non-invading cells; migrated or invaded cells were fixed, stained, and counted under the microscope (Olympus, Japan).

### MTS Assay

Cell proliferation was measured at 24, 48, and 72 h after transfection in 96-well plates (Corning, NY, USA). First, 20 μl of 3-(4,5-dimethylthiazol-2-yl)-5-(3-carboxymethoxyphenyl)-2-(4-sulfophenyl)-2H-tetrazolium (MTS) solution (5 mg/ml) was added to the culture medium in each well, and absorbance was read at 490 nm using a microplate reader (Bio-Rad, Foster City, CA, USA).

### EdU Assay

The transfected gastric cancer cells were seeded into 96-well plates for use. Proliferation of cells was also assessed by a Cell-Light™ EdU DNA Cell Proliferation (RiboBio, Guangzhou, China) assay according to the manufacturer's instructions. Gastric cancer cells were treated with DAPI (mark nucleus) after the 5-ethynyl-2′-deoxyuridine (EdU) incubation and counted under a fluorescent microscope (Olympus, Japan).

### Apoptosis Assay

Gastric cancer cells were obtained 48 and 72 h after transfection and were stained using Annexin V-FITC/PI Apoptosis Detection Kit (BestBio, Shanghai, China) according to the manufacturer's instructions and immediately analyzed by flow cytometry.

### Western Blot

Western blot was performed according to standard methods. Proteins were collected 48 h after transfection. Primary antibodies used included h-Ras (1:1,000, Sangon Biotech), Raf-1 (1:1,000, Cell Signaling), mitogen-activated protein kinase/extracellular signal-regulated kinase (MEK) (1:1,000, Cell Signaling), extracellular signal-regulated kinase (ERK) (1:2,000, Cell Signaling), p90RSK (1:1,000, Cell Signaling), AKT (1:10,000, Abcam), AKT3 (1:2,000, Proteintech), PIK3-CD (1:2,000, Proteintech), and β-actin (1:1,000, OriGene). The antibody binding was detected using a FluorChem Q system (Cell Biosciences, San Jose, CA, USA).

### Statistical Analysis

All statistical analyses were performed using GraphPad Prism 5 (GraphPad Software, Inc., San Diego, CA, USA). The significance of the differences was determined by the Student's *t*-test between two groups. Non-parametric test was used in calculating RNA expression in samples. The chi-square test was used to analyze the relationship between clinicopathological factors and AK025387 expression. The receiver operating characteristics (ROC) curve was used to measure the diagnostic value of AK025387 expression in gastric cancer. The survival analysis was assessed by the Kaplan–Meier method, and the differences between groups were examined by the log-rank test. In this study, all *in vitro* experiments were repeated for at least three times. *P* < 0.05 was considered to be statistically significant.

## Results

### LncRNA AK025387 Is Upregulated in Metastatic Gastric Cancer Sample

**Figure 1:**
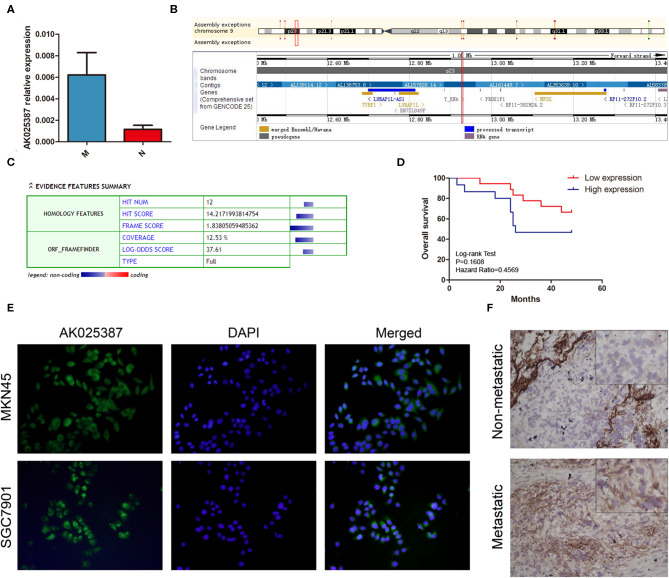
Expression of AK025387 in gastric cancer tissues and intracellular location of AK025387. **(A)** The relative expression of AK025387 was higher in the metastatic (M) group than that in the non-metastatic (N) group (*P* = 0.010). **(B)** Gene information of AK025387. AK025387 is transcribed on chromosome 9 p23, with a length of 1,892 nucleotides, containing two exons and one intron. **(C)** Coding Potential Calculator (CPC) results of AK025387 (coding potential score: 0.024, weak coding potential). **(D)** Correlation of AK025387 expression with survival of patients with gastric cancer. No significant correlation was observed between overall survival and AK025387 expression (*P* = 0.161). **(E)** Fluorescence *in situ* hybridization results of AK025387 in gastric cancer cell lines. AK025387 was located in both nucleus and cytoplasm, but mainly in cytoplasm in both MKN45 and SGC7901 cell lines. **(F)** The *in situ* hybridization results of AK025387 in metastatic and non-metastatic gastric cancer tissues (200×). AK025387 was located mainly in the cytoplasm.

In our previous study, we had assessed the expression profiles of 33,045 lncRNAs through microarray analysis on five gastric cancer samples with lymphatic metastasis and five gastric cancer samples without lymphatic metastasis (array data were available in the GEO database, GSE72307; Probe name, ASHG19A3A038370). The result revealed that lncRNA, AK025387, was upregulated in metastatic gastric cancer sample with a 2.9-fold change (*P* = 0.04). To verify the microarray result, qRT-PCR was used to test AK025387 relative expression in both metastatic and non-metastatic gastric cancer samples (37 in metastatic group, 33 in non-metastatic group). The relative expression of AK025387 was higher in metastatic group than that in non-metastatic group (*P* = 0.010, Figure 1A), which was consistent with the microarray result. Furthermore, we analyzed the correlation between AK025387 expression and clinicopathological features in metastatic and non-metastatic gastric cancer (Table 1). A ROC curve was used to evaluate the AK025387 expression in predicting metastasis and non-metastasis (sensitivity = 69.0%, specificity = 73.3%, *P* = 0.008). AK025387 expression was found to be positively correlated with lymph node metastasis (*P* = 0.025). No significant correlation was observed between AK025387 expression and age, gender, tumor size, depth of invasion, and differentiation (*P* = 0.763, *P* = 0.700, *P* = 1.000, *P* = 0.369, *P* = 1.000, respectively). This result indicates a strong relationship with gastric cancer metastasis but not other clinicopathological factors. Furthermore, we evaluated the association of AK025387 expression with the prognosis of gastric cancer patients. However, the survival analysis indicates no significant correlation between overall survival and AK025387 expression (*P* = 0.161, Figure 1D).

**Table 1:** Association between AK025387 expression and clinicopathological factors in primary gastric cancer.

**Variable**	***N***	**AK025387 expression**		***P*-value**
		**Low**	**High**	
**AGE**				
≤ 60	23	9	14	
>60	20	9	11	
Missing	3	1	2	0.763
**GENDER**				
Male	38	15	23	
Female	8	4	4	0.700
**TUMOR SIZE**				
≤ 4	17	7	10	
>4	24	10	14	
Missing	5	2	3	1.000
**DEPTH OF INVASION**				
T1	1	0	1	
T2	6	4	2	
T3	16	7	9	
T4	22	7	15	0.369
Missing	1	1	0	
**LYMPH NODE METASTASIS**				
Negative	15	10	5	
Positive	31	9	22	0.025
**DIFFERENTIATION**				
Well	0	0	0	
Moderate	8	3	5	
Poor	35	13	22	
Missing	3	3	0	1.000

Next, we used UCSC Genome Browser (http://genome.ucsc.edu/) to search genetic information of AK025387 (Figure 1B). AK025387 is transcribed on chromosome 9 p23, with a length of 1,892 nucleotides. Coding Potential Calculator (https://opendata.pku.edu.cn/dataset.xhtml?persistentId=10.18170/DVN/8BO9C9) (13) was used to estimate protein-coding potential. The result illustrated that AK025387 had a low coding potential (Figure 1C). In order to investigate the subcellular localization of AK025387, we designed *in situ* hybridization in gastric cancer cells and tissues. The result showed that AK025387 was located in both nucleus and cytoplasm, mostly in cytoplasm (Figures 1E,F). This finding may demonstrate the potential function of AK025387 in gastric cancer cell.

### AK025387 Promotes Migration and Invasion of Gastric Cancer Cell *in vitro*

**Figure 2:**
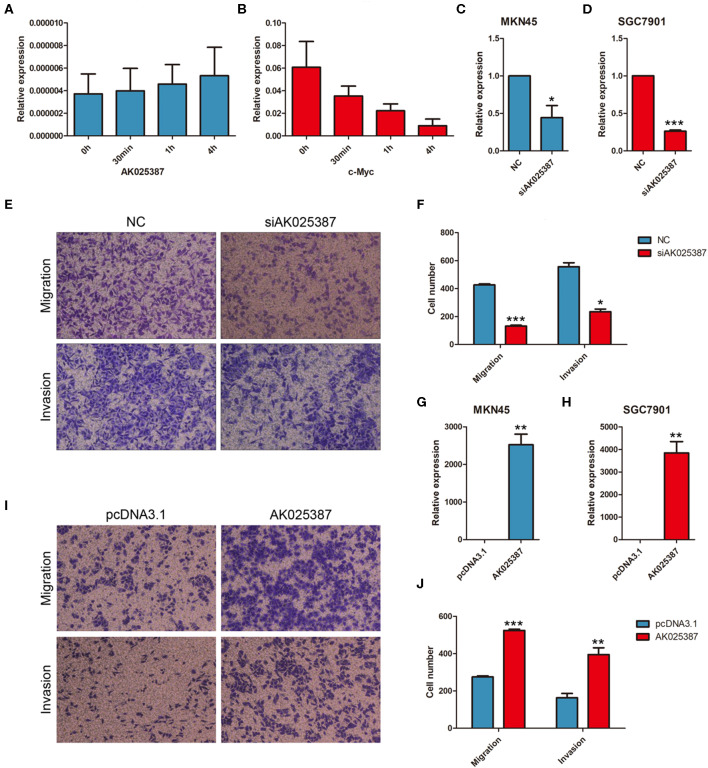
AK025387 promoted migratory and invasive ability of gastric cancer cells. Actinomycin D could inhibit the synthesis of RNA. AK025387 maintained its expression level in MKN45 after treated **(A)** with Actinomycin D **(B)** compared with c-myc. **(C,D)** The small interfering RNA (siRNA) could sufficiently downregulate the expression level of AK025387 in both MKN45 and SGC7901 cell lines (*P* = 0.038 in MKN45; *P* < 0.001 in SGC7901). **(E,F)** Transwell experiments showed an inhibition of migration (*P* < 0.001) and invasion (*P* = 0.010) with knockdown of AK025387 in MKN45 cell line. **(G,H)** Upregulation of AK025387 in MKN45 and SGC7901 cell lines (*P* = 0.006 in MKN45; *P* < 0.009 in SGC7901). **(I,J)** The overexpression of AK025387 promoted migration (*P* < 0.001) and invasion (*P* = 0.007) in MKN45 cell line. *P < 0.05, **P < 0.01, and ***P < 0.001.

To evaluate the biological functions of AK025387 *in vitro*, we chose two gastric cancer cell lines, MKN45 and SGC7901, for further experiments (Supplementary Figure 4). RNA stability assay was used to test the stability of AK025387 in gastric cancer cells. Compared with c-myc, AK025387 expression was stable in 0 h, 30 min, 1 h, and 4 h in MKN45 (Figures 2A,B). The same results were also seen in other gastric cancer cell lines (data not shown). Then, a small interfering RNA (siRNA) was designed to knock down AK025387 in MKN45 and SGC7901 (*P* = 0.038 in MKN45; *P* < 0.001 in SGC7901, Figures 2C,D). Transwell experiments were performed to investigate migratory ability of MKN45 and SGC7901 cells. The migratory ability was inhibited with the silence of AK05387 in both MKN45 and SGC7901 cell lines, which means that AK025387 may promote migration of gastric cancer cells (*P* < 0.001 in MKN45; *P* = 0.005 in SGC7901, Figures 2E,F; Supplementary Figures 1A,B). In addition, we tested invasive ability of MKN45 and SGC7901 cells using Matrigel-coated Transwell experiments to determine whether AK025387 could promote invasive behavior or not. The invasive ability was inhibited in MKN45 and SGC7901 cell lines with downregulated AK025387 (*P* = 0.010 in MKN45; *P* = 0.004 in SGC7901. Figures 2E,F for MKN45; Supplementary Figures 1A,B for SGC7901).

Then, AK025387 was overexpressed in MKN45 and SGC7901 cell lines (*P* = 0.006 in MKN45; *P* = 0.009 in SGC7901, Figures 2G,H). The migratory and invasive abilities were enhanced with the upregulation of AK05387 in MKN45 cell line (*P* < 0.001 in migration; *P* = 0.007 in invasion, Figures 2I,J). An increased tendency but not significant difference of migration and invasion was observed in SGC7901 cell line with AK025387 upregulated (*P* = 0.336 in migration; *P* = 0.081 in invasion, Supplementary Figures 1C,D). Altogether, these results confirm that AK025387 could promote gastric cancer cell migratory and invasive ability.

### AK025387 Promotes Growth of Gastric Cancer by Inhibiting Apoptosis *in vitro*

**Figure 3:**
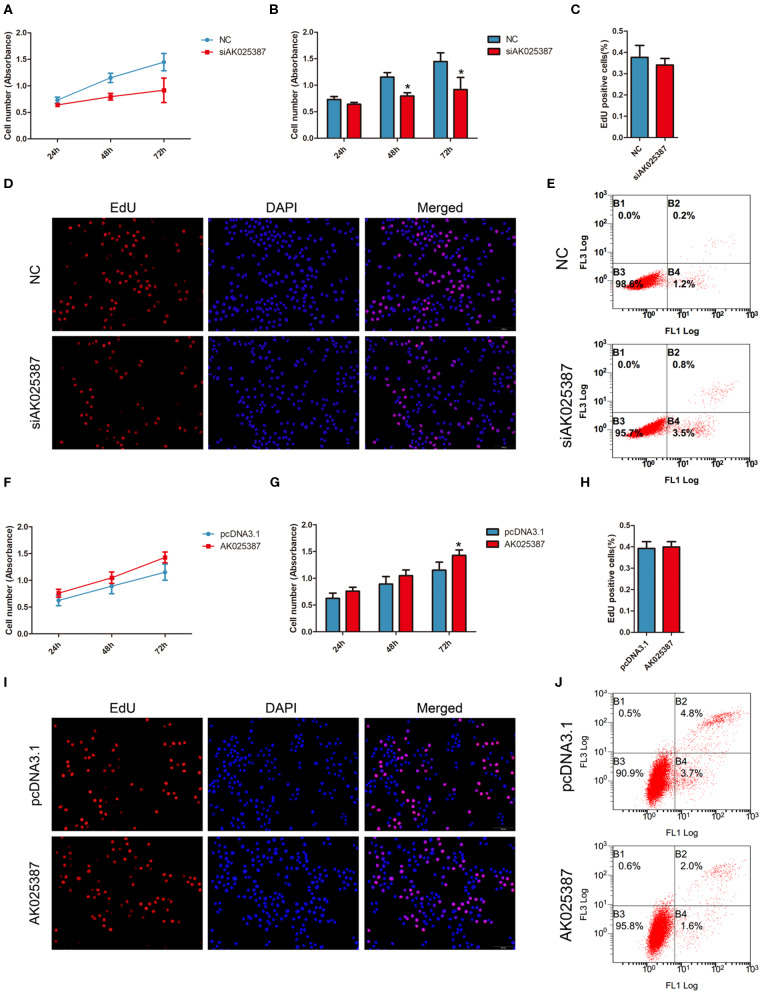
AK025387 promoted gastric cancer growth via inhibiting apoptosis *in vitro*. **(A,B)** 3-(4,5-Dimethylthiazol-2-yl)-5-(3-carboxymethoxyphenyl)-2-(4-sulfophenyl)-2H-tetrazolium (MTS) assay showed an inhibition of growth ability with AK025387 knockdown in MKN45 cell line. The absorbance of siAK025387 group had no difference with the control group in 24 h (*P* = 0.082) but was lower in 48 h (*P* = 0.020) and 72 h (*P* = 0.017) in MKN45 cell line. **(C,D)** The 5-ethynyl-2′-deoxyuridine (EdU) assay showed no difference between the control and siAK025387 groups in MKN45 (*P* = 0.352). **(E)** Flow cytometry indicated a higher apoptosis rate with AK025387 knockdown in MKN45 cell line. FL1: Annexin V-FITC. FL3: PI. **(F,G)** The overexpression of AK025387 promoted growth ability in the MKN45 cell line. The absorbance of AK025387-upregulated group was higher in 72 h (*P* = 0.020) but not in 24 h (*P* = 0.090) and 48 h (*P* = 0.051) in MKN45. **(H,I)** The EdU assay showed no difference between the control and pcDNA3.1-AK025387 groups in MKN45 (*P* = 0.408). **(J)** Upregulation of AK025387 inhibited apoptosis in the MKN45 cell line. FL1: FITC. FL3: PI. *P < 0.05.

To investigate the effect of AK025387 on growth, MTS assay was performed in both MKN45 and SGC7901 cell lines. An inhibition of cell growth ability was shown in AK025387-downregulated expression group (Figures 3A,B; Supplementary Figures 2A,B). To verify this result, cell proliferation ability was further detected via EdU assay. However, we found that there was no difference between control and AK025387-downregulated group in two gastric cancer cell lines (*P* = 0.352 in MKN45; *P* = 0.402 in SGC7901, Figures 3C,D; Supplementary Figures 2C,D). As cancer cell growth involved proliferation and apoptosis, we detected the effect of AK025387 on the apoptosis rate of gastric cancer cell lines. The result indicates that both MKN45 and SGC7901 cell lines transfected with AK025387 siRNA show a tendency of higher apoptosis ability compared with control group (Figure 3E; Supplementary Figures 2E, 5A,C).

Next, we overexpressed AK025387 in MKN45 and SGC7901 cell lines. A promotion of cell growth ability was observed in both cell lines with AK025387 upregulated by MTS assay (Figures 3F,G; Supplementary Figures 2F,G). No significant differences were found between control and AK025387-upregulated group by EdU assay (*P* = 0.408 in MKN45; *P* = 0.247 in SGC7901, Figures 3H,I; Supplementary Figures 2H,I). In addition, a lower apoptosis tendency was observed in AK025387-upregulated group in both cell lines (Figure 3J; Supplementary Figures 2J, 5B,D). All the above results suggests that AK025387 promotes growth of gastric cancer by inhibiting apoptosis without promoting proliferation directly.

### AK025387 Performs Its Biological Function via MAPK Pathway

**Figure 4:**
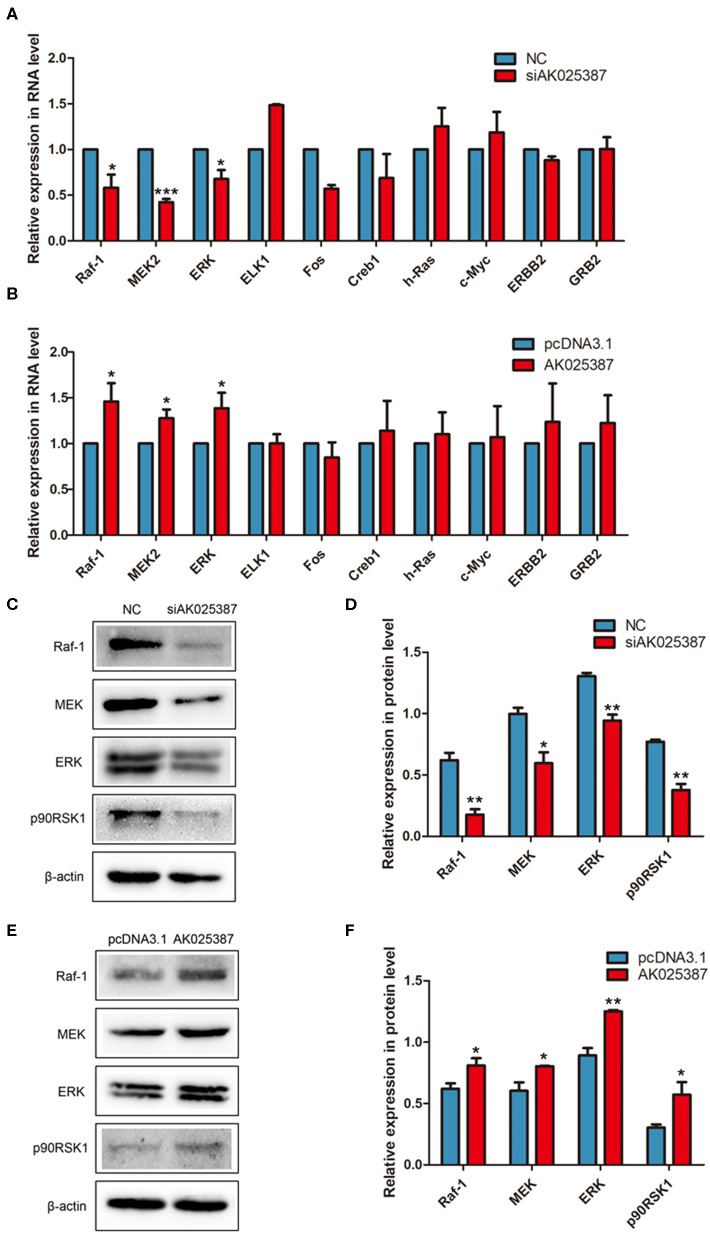
AK025387 was involved in mitogen-activated protein kinase (MAPK) signaling pathway. **(A,B)** The expression levels of some RNAs in MAPK pathway in the MKN45 cell line. **(A)** Raf-1 (*P* = 0.022), MEK2 (*P* < 0.001), and ERK (*P* = 0.015) were significantly downregulated with knockdown of AK025387. **(B)** Raf-1 (*P* = 0.042), MEK2 (*P* = 0.023), and ERK (*P* = 0.043) were significantly upregulated with overexpression of AK025387. **(C–F)** The Western blot results of some proteins in MAPK pathway in the MKN45 cell line. **(C,D)** Raf-1, MEK, ERK, and p90RSK1 were significantly downregulated with knockdown of AK025387 (*P* = 0.004, *P* = 0.018, *P* = 0.003, *P* = 0.002, *P* = 0.001, respectively). **(E,F)** Raf-1, MEK, ERK, and p90RSK1 were significantly upregulated with overexpression of AK025387 (*P* = 0.031, *P* = 0.023, *P* = 0.002, *P* = 0.032, *P* = 0.001, respectively). *P < 0.05, **P < 0.01, and ***P < 0.001.

To explore how AK025387 regulated migration and invasion of gastric cancer cell, we first focused on the expression change of neighboring gene of AK025387, but no significant association was observed as AK025387 downregulated (data not shown). Then, several classical signaling pathways were tested via qRT-PCR and Western blot analysis. MAPK pathway mediated invasion and proliferation in many types of human cancers (14), and lncRNAs might be involved in this signaling pathway. Several genes in MAPK pathway were tested in MKN45 and SGC7901 gastric cancer cell lines with different levels of AK025387. The messenger RNA (mRNA) expression levels of Raf-1, MEK2, and ERK showed a parallel change with AK025387 downregulation (Figure 4A, *P* = 0.022, *P* < 0.001, *P* = 0.015, respectively) and AK025387 upregulation (Figure 4B, *P* = 0.042, *P* = 0.023, *P* = 0.043, respectively). Additionally, Western blot was performed to confirm this result at protein level (Figures 4C-F). In addition, we tested several representative genes in the PI3K-AKT pathway, but no significant difference was observed between the AK025387 and PI3K-AKT pathway ((Supplementary Figures 3A-C). All the above results imply that AK025387 may play a role in MAPK signaling pathway to perform its biological function.

## Discussion

As gastric cancer is one of the most common carcinomas worldwide, it is essential to research on the mechanism of gastric cancer occurrence and development. LncRNA is one of the indispensable molecules involved in this process. Zhang et al. identified the lncRNA HOXC-AS3 that regulated gastric cancer cell proliferation and migration via interacting with YBX1 (15). LncRNA ZFPM2 antisense RNA 1 (ZFPM2-AS1) was reported to promote proliferation and suppress apoptosis of gastric cancer cells via a novel ZFPM2-AS1/MIF/p53 signaling axis (16). Besides, lncRNA metastasis-associated lung adenocarcinoma transcript 1 (MALAT1) was overexpressed in gastric cancer cells and promote cell proliferation in gastric cancer by recruiting SF2/ASF (17). Another famous lncRNA HOTAIR was also associated with gastric cancer (12). HOTAIR might act as competing endogenous RNA and interact with microRNA (miRNA), such as miR-331-3p and miR-152, to regulate human epidermal growth factor receptor 2 (HER2) and human leukocyte antigen-G (HLA-G) expression in gastric cancer cells (12, 18–20).

Since most lncRNAs were found between cancer and non-cancerous tissue in gastric cancer, there was a lack of attention on the aberrant lncRNAs between metastatic and non-metastatic cancer. We thus focused on this area, which might be more significant in metastasis and invasion of tumor. In this study, we validated that AK025387 expression was significantly increased in metastatic gastric cancer sample compared with the non-metastatic gastric cancer samples. The clinicopathological analysis elaborated a strong relationship between AK025387 expression and gastric cancer metastasis, ruling out other factors. However, no correlation was observed between overall survival of gastric cancer patients and AK025387 expression. We also testified that AK025387 was located in both nucleus and cytoplasm, but mainly in cytoplasm. Then, the expression of AK025387 was downregulated in gastric cancer cell lines using siRNA transfection, and a reduction in migration and invasion was found. Interestingly, the migratory and invasive ability was significantly promoted with AK025387 upregulated in MKN45 but not in SGC7901 cell lines. We consider that SGC7901 cell line represents gastric cancer cells with limited migration and invasion due to AK025387 upregulation. Thus, the enhancement of migration and invasion by upregulating AK025387 in SGC7901 is limited.

The function of AK025387 in proliferation and apoptosis was also investigated. Different results performed by MTS and EdU assays indicated that AK025387 promoted growth of gastric cancer in another way. An apoptosis assay was explored, and it was demonstrated that AK025387 promoted growth of gastric cancer by inhibiting apoptosis but not promoting proliferation directly. Additionally, some apoptosis-relative markers were tested [such as BCL2 and BCL2-antagonist of cell death (BAD), data not shown], and no significance was found. More researches on the mechanism of apoptosis are still required.

MAPK cascades regulate several cellular processes such as cell proliferation, differentiation, metabolism, motility, survival, apoptosis, etc. and contribute to many physiological and pathological processes (21). The ERK pathway is one of the most well-known MAPK pathways in mammals. The ERK is activated upon phosphorylation by MEK, which is itself activated when phosphorylated by Raf (22). The ERK/MAPK pathway is also one of the most frequently affected pathways in cancer (23, 24). In recent years, more researches have proposed that lncRNAs regulate signaling pathways in cancer, including ERK/MAPK pathway (25). It is reported that lncRNA MALAT-1 inactivates ERK/MAPK pathway to mediate tumor suppression in glioma cells (26) and may promote the proliferation and metastasis of gallbladder cancer cells by activating the ERK/MAPK pathway (27). LncRNA CCHE1 (cervical carcinoma expressed PCNA regulatory lncRNA) promoted carcinogenesis and indicated a poor prognosis of hepatocellular carcinoma via activation of ERK/MAPK pathway (28). In this study, we discovered a reduction in the expression level of Raf-1, MEK2, and ERK with AK025387 knockdown. However, some upstream and downstream molecules in MAPK pathway, such as growth factor receptor-bound protein 2 (GRB2), c-myc, and Erb-B2 receptor tyrosine kinase 2, showed no expression level change with AK025387 interfered. These results indicate that AK025387 might activate the Raf-MEK-ERK pathway to promote migration and invasion and inhibit apoptosis of gastric cancer cells, but a specific signaling pathway to regulate its biological function is still unknown. Further research on molecular mechanism of AK025387 in gastric cancer, such as direct or indirect interaction with MAPK pathway, is still needed.

## Conclusions

In summary, we find that AK025387 is significantly upregulated in metastatic gastric cancer samples. The expression of AK025387 is confirmed to be significantly positively correlated only with lymph node metastasis and is not associated with overall survival of gastric cancer patients. AK025387 is located in both nucleus and cytoplasm, but mainly in cytoplasm. AK025387 promotes gastric cancer cells' migratory and invasive ability but inhibited apoptosis *in vitro*. Moreover, AK025387 increases the expression level of Raf-1, MEK2, and ERK. Therefore, AK025387 may activate the Raf-MEK-ERK pathway to perform its biological function. Taken together, AK025387 is a potential molecule and needs to be further investigated to determine if it can be used as a biomarker of metastatic gastric cancer.

## Data Availability Statement

The datasets generated for this study can be found in the GEO database (GSE72307).

## Ethics Statement

The studies involving human participants were reviewed and approved by Ethical Committee of Shandong University, China. The patients/participants provided their written informed consent to participate in this study.

## Author Contributions

Y-YS, HZ, R-RM, and PG designed the study. Y-YS, G-HZ, Y-RT, and LeL performed the experiments. LiL collected the gastric cancer samples. Y-YS analyzed the data and wrote the manuscript. All authors read the manuscript and approved the final draft that was submitted.

## Conflict of Interest

The authors declare that the research was conducted in the absence of any commercial or financial relationships that could be construed as a potential conflict of interest.
