# Supplementary material for: Long Non-coding RNA AK025387 Promotes Cell Migration and Invasion of Gastric Cancer
Source: Front Oncol. 2020 May 20;10:633. doi: 10.3389/fonc.2020.00633 (PMC7251172; doi:10.3389/fonc.2020.00633)
Supplement: Supplementary file 1 [file Data_Sheet_1.doc]

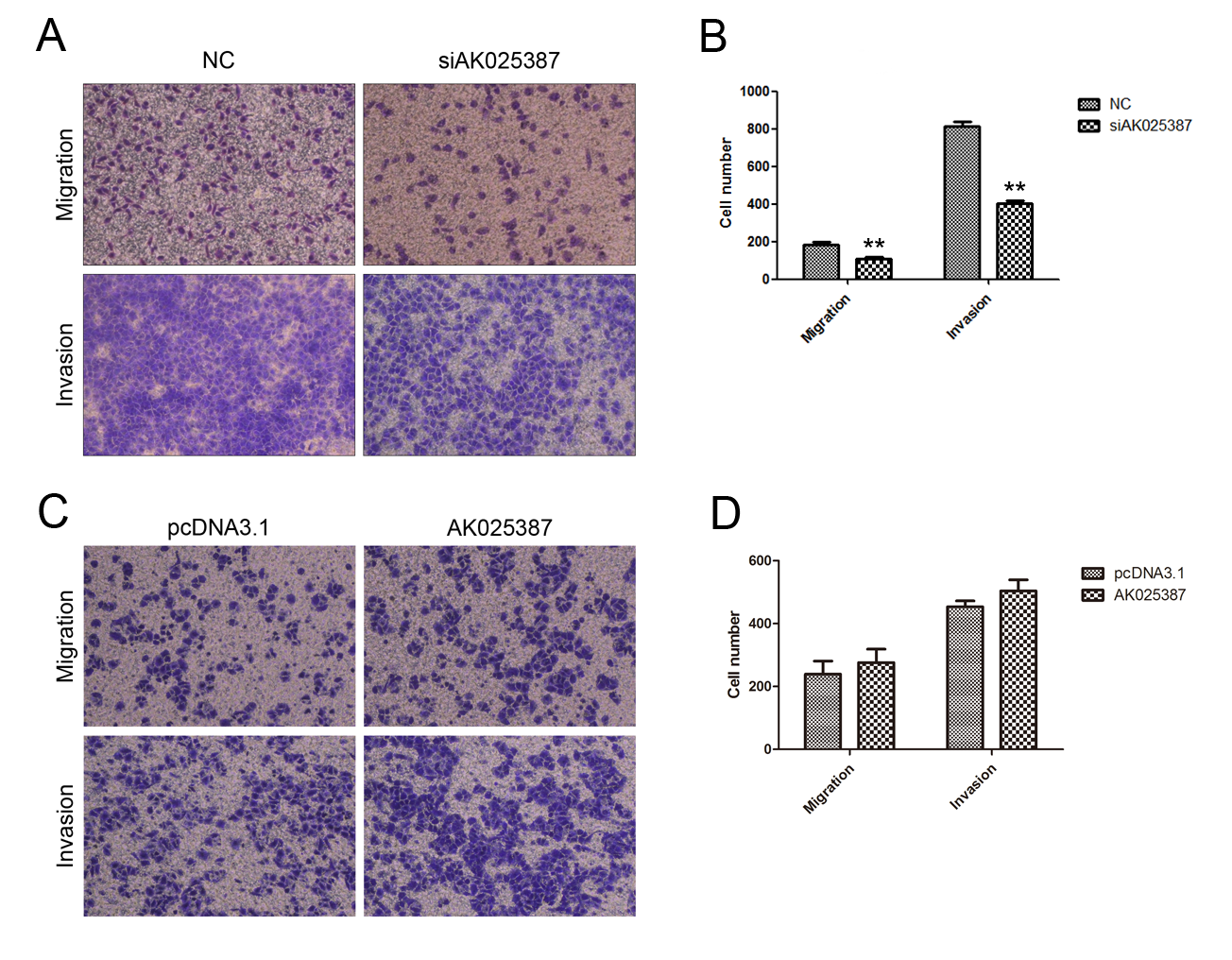


**Supplementary Figure 1:** **AK025387 promoted metastatic and invasive ability of gastric cancer cells in SGC7901 cell line.** (A, B) Transwell experiments showed an inhibition of migration (*P*=0.005) and invasion (*P*=0.004) with knockdown of AK025387 in MKN45 cell line. (C, D) An increased tendency but not significant difference of metastasis and invasion was observed in SGC7901 cell line with AK025387 upregulated (*P*=0.336 in metastasis; *P*=0.081 in invasion). **: *P*<0.01.


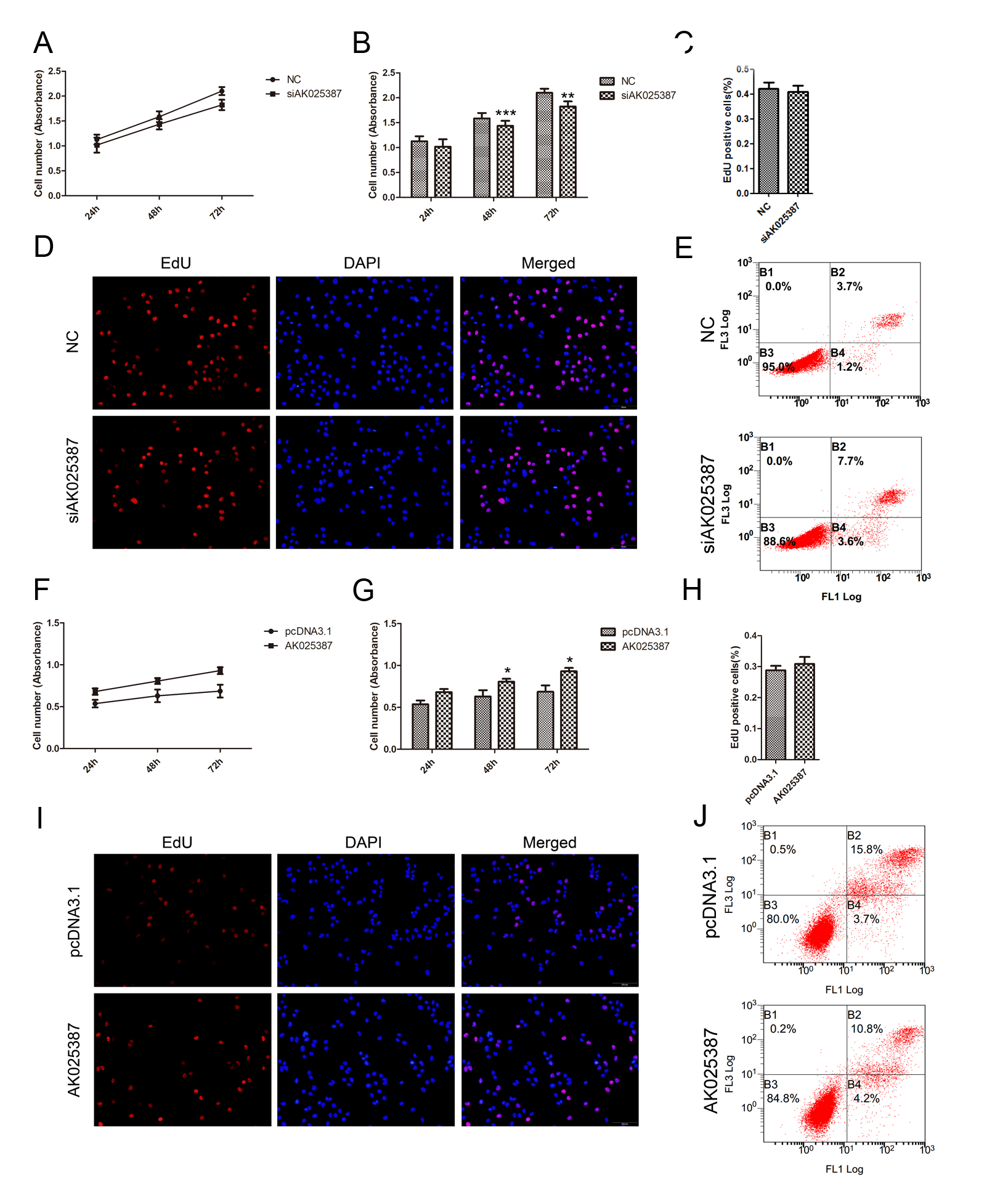


**Supplementary Figure 2: AK025387 promoted gastric cancer growth via inhibiting apoptosis in SGC7901 cell line.** (A, B) MTS assay showed an inhibition of growth ability with AK025387 knockdown at 48 hours and 72 hours in SGC7901 cell line (*P*= 0.169 at 24 hours; *P*=0.001 at 48 hours; *P*=0.028 at 72 hours). (C, D) The EdU assay showed no difference between control group and siAK025387 group in SGC7901 (*P*=0.402). (E) Flow cytometry indicated a higher apoptosis rate with AK025387 knockdown in SGC7901 cell line. FL1: Annexin V-FITC. FL3: PI. (F, G) The overexpression of AK025387 promoted growth ability at 48 hours and 72 hours in SGC7901 cell line (*P*=0.084 at 24 hours; *P*=0.05 at 48 hours; *P*=0.024 at 72 hours). (H, I) The EdU assay showed no difference between control group and pcDNA3.1-AK025387 group in SGC7901 (*P*=0.247). (J) Upregulation of AK025387 inhibited apoptosis in SGC7901 cell line. FL1: FITC. FL3: PI. *: *P*<0.05, **: *P*<0.01, ***: *P*<0.001.


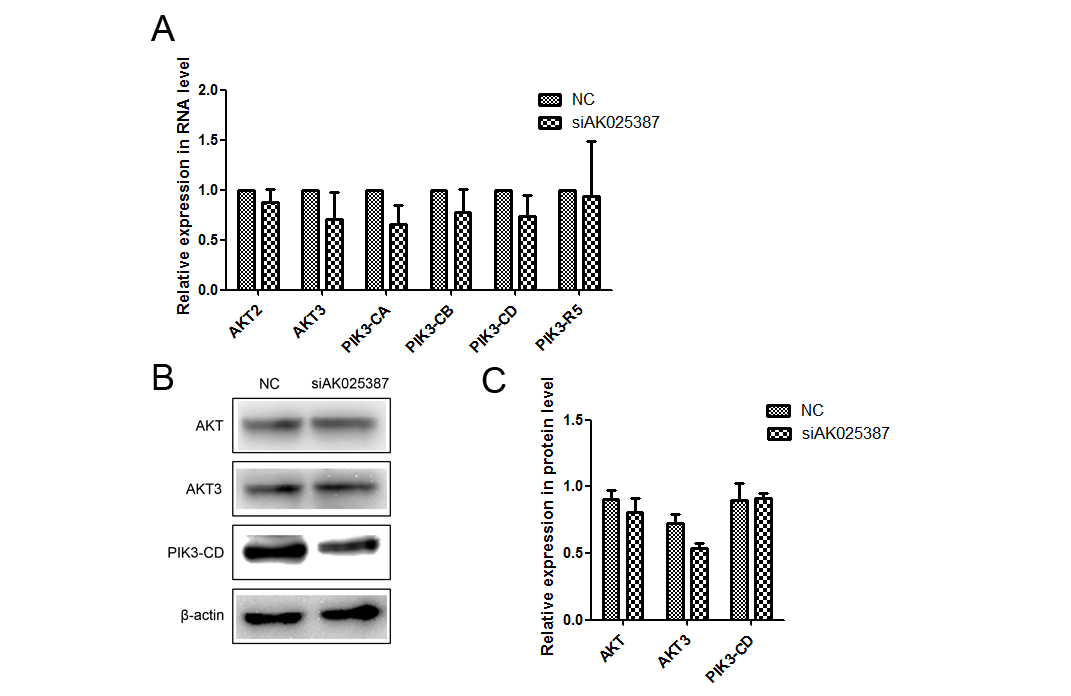


**Supplementary Figure 3: AK025387 was not involved in PI3K-AKT signaling pathway.** (A) The expression level of some RNAs in PI3K-AKT pathway in MKN45 cell line. No significant differences were found between control and siAK025387 group. (B, C) The western blot results of some proteins in PI3K-AKT pathway in MKN45 cell line. Level of some proteins in PI3K-AKT pathway had no differences between control and siAK025387 group.


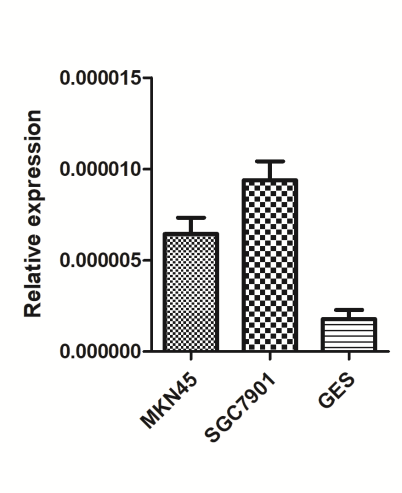


**Supplementary Figure 4: Relative expression of AK025387 to GAPDH in MKN45, SGC7901 and GES.** The expression of AK025387 in gastric cancer cells was higher than that in GES cell line.


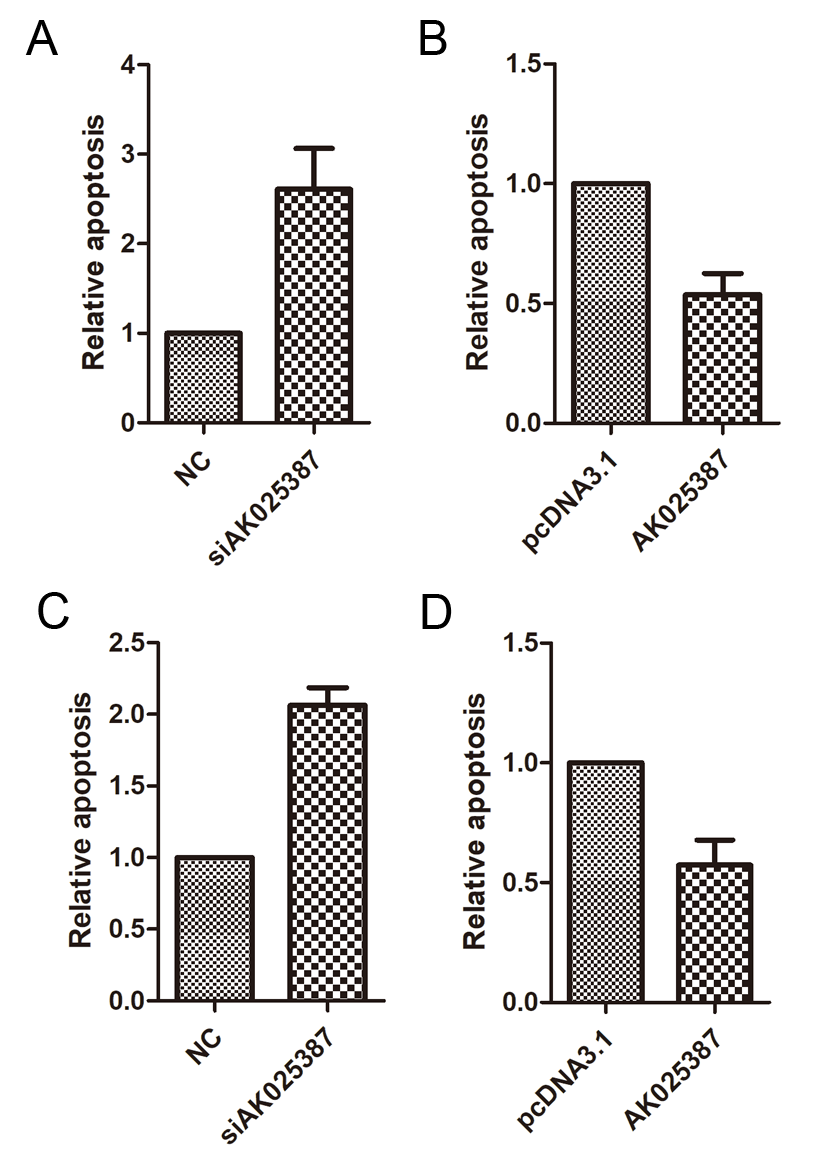


**Supplementary Figure 5: Relative apoptosis based on the results of flow cytometry.** (A, B) Relative apoptosis in MKN45 cells. (C, D) Relative apoptosis in SGC7901 cells.
